# Rethinking temporalities through being with children: A dialogic reflection

**DOI:** 10.1177/20436106261449427

**Published:** 2026-05-25

**Authors:** Markéta Supa, Rebekah Willett, Kristiina Kumpulainen, Harini Rajagopal, Jenny Byman, Layla Coleman, Jacqueline Kociubuk, Jenny Renlund, Chin Chin Wong, Melanie Wong

**Affiliations:** 1 Charles University, Prague, Czech Republic; 2 University of Wisconsin-Madison, WI, USA; 3 University of British Columbia, Vancouver, BC, Canada; 4 University of Helsinki, Finland; 5 University of Rhode Island, Kingston, RI, USA

**Keywords:** children, temporality, slow scholarship, (post-)qualitative research

## Abstract

Children’s everyday lives are shaped by shifting rhythms of movement, suspension, and retrospection through which time becomes lived, imagined, and meaningful. This article brings together interdisciplinary and international perspectives, with members of a collective reflecting on temporality in children’s lives. Across a prologue and nine reflective pieces developed through prolonged dialogue, the article explores time in children’s diverse everyday contexts — including home, school, public, digital, and natural environments — in Canada, Czechia, Finland, and the USA. By applying a temporal lens to projects spanning literacy, ecology, placemaking, and media, the collection conceptualises time as non-linear, plural, situational, and relational. This perspective on time and childhood enables a closer understanding of how children’s experiences unfold within tensions between progress-oriented social expectations, ecological timeframes, and their own ways of being and learning. It also highlights the importance of slow and stretched temporalities for creative, reflective, playful, and imaginative practices. By foregrounding dialogue and deep listening, the article promotes responsive and empathetic approaches to research with children. Developed through collaborative practices grounded in an ethics of care, it also manifests slow childhood scholarship.

## Prologue: Towards slow childhood scholarship

### Markéta Supa and Rebekah Willett

Viewing children’s lives and childhood research through a temporal lens illuminates the depth and breadth of children’s experiences (Gavin, 2022). It also unsettles familiar binaries that continue to shape how childhood is understood (Uprichard, 2008), for example, children as beings or becomings, childhood as lived experience or conceptual construct, mind and body, adult and child, and human and non-human. Against this backdrop, the relationship between childhoods and time has attracted increasing scholarly attention (e.g. Pomerantz and Knight, 2025). The present collective joins and extends these discussions by foregrounding children as whole beings who experience, imagine, and make meaning of time subjectively, yet always in relation to spaces, materials, and other human and more-than-human beings. We engage in “thinking time differently” (Barad, 2018) through dialogic reflection on our research in the fields of literacy, media, and education across various environments (home, school, public, digital, and rural) in Canada, Czechia, Finland, and the USA. Approaching time as moving backwards, forwards, and sideways, and as lived through moments of stillness, pause, and elasticity, we ultimately invite temporally sensitive ways of being with children in research and everyday life.

The article is framed as a series of nine diverse yet interconnected contributions developed through sustained dialogue within the *Children and Placemaking Collective*, an informal international network of scholars committed to slow and collaborative childhood scholarship. Our devotion to this form of scholarship required careful reading of selected texts, conversations over many months, and different forms of engagements with ideas including writing personal reflections and poetry. Such slow reading and writing stands in contrast with many of our experiences of rushing through journal articles to locate “just-in-time” references in order to support ideas about which we were writing.

Our slow childhood scholarship, however, is not only resistance to the neoliberalisation of universities that require high levels of “productivity” as discussed by numerous academics (e.g. Berg and Seeber, 2016; Hartman and Darab, 2012). Additionally, resisting the pressure to always publish new findings, we recognise that our research with children improves when we revisit our data, taking time to think more deeply, listen more closely, and to develop new frameworks for analysis and so on. This article, therefore, represents a commitment to processes of research that are best approached slowly. Further, in line with feminist politics, our approach involves a commitment to an ethics of care for fellow scholars. Our time together provided us new ways to “recognize the value of collective authorship, mentorship, collaboration, community building, and activist work in the germination and sharing of ideas” (Mountz et al., 2015: 1250).

Inspired by earlier collective explorations by Da Rosa Ribeiro et al. (2023) that seek to release childhood from the hegemony of linear, sequential, and progressive time, our colloquium likewise questions dominant temporal understandings that shape today’s childhoods. Yet rather than focusing on conceptual, philosophical, and historical thinking about childhood, our contributions explore temporalities in concrete moments of being with children and children being with others. As a collection, the article offers an interdisciplinary synthesis of affective, relational, and situational perspectives on children and time. We explore how children live and imagine time while learning, making, playing, storying, reading, and engaging with media across diverse contexts. Interwoven throughout is a reflection on our own positioning as qualitative researchers. We rethink interactions with children as temporal spaces for pause, dialogue, and justice, while fostering more responsive and empathetic approaches to childhood research.

The first three reflective pieces by Jenny R, Kristiina, and Chin Chin with Jenny B. are situated within interrelated research projects that draw on posthuman, decolonial, and new materialist perspectives. These contributions dwell on children’s environmental and ecological experiences and learning, while exploring cyclical and rhythmical understandings of time within multispecies and more-than-human relations. The following triad, by Jacquie, Layla, and Melanie, interrogates power and agency related to time in diverse spatial and embodied contexts: self-care children in libraries, rural childhoods, and school and online learning environments. These reflections explore children’s perceived spatialities and their negotiation, allocation, and meaning making of time. While experiential approaches to time are present across the collection, the reflections by Rebekah, Markéta, and Harini accentuate this perspective, deepening the critique of hurried and forward-driven temporalities. Emphasis is placed on the importance of slow and expansive time for creative, reflective, playful, and imaginative practices. Attending to children as aware of their past, present, and future selves—and to researchers as needing to be attuned to these temporal experiences, as well as to questions of equality and marginalisation—these reflections highlight the value of listening to children’s media experiences and personal stories.

Foregrounding temporality as a shared reflective lens, this collective exploration contributes to ongoing debates in global childhood studies concerning the relational and contextually shaped nature of children’s lives. At the same time, it highlights the potential of slow childhood scholarship to foster respectful and care-orientated dialogue across disciplines, perspectives, and geographical locations.

## Children’s environmental experiences and imaginaries as disruptions to Western educational timeframes

### Jenny Renlund

Linear and abstracted notions of time, positioned as a separate tool for structuring everyday life and for measuring efficiency and progress along a continuum, are central to many educational systems and discourses (Da Rosa Ribeiro et al., 2023; Gavin, 2022). This notion of time positions the learning human as an individualised thinking mind and performs dualistic separations such as body/mind and space/time (Stirling, 2022). Here, I draw inspiration from a story my daughter shared with me when she was 10 years old, to consider how children’s temporal landscapes and imaginaries might provoke reconfigurations of time in education.

My daughter’s biggest fear is to live forever. She finds the thought of everything else on earth gradually dying and going extinct and herself remaining alive and unchanged terrifying. She imagines herself in a distant future, invulnerable and floating around in dark space, when earth has ‘finally’ been destroyed.

My daughter’s fear touches on the finitude of earth. When exploring children’s (aged 7–9) ecological imagination with my colleagues, we have encountered similar narratives from other children, who describe looming futures of the earth exploding and local landscapes being flooded or ravaged by pollution and waste (Byman et al., 2022; Kumpulainen et al., 2023; Renlund et al., 2022). Such narratives and imaginaries make evident the tensions of children’s everyday lives and highlights how educational time performs a contradiction to the actual material becoming of places and human bodies as dynamic, earthly and relational. My daughter’s vision can easily be connected to a future of climate change and ecological destruction. Simultaneously, this vision of the future touches on the material-temporal paradox surrounding the invulnerable, individual adult personhood, which the enlightenment discourse in Western educational systems celebrates as the end goal of childhood (Stirling, 2022). My daughter’s story makes tangible the absurd existence of such an independent, timeless and materially disembodied creature. Within the fear of being a static entity in a changing universe, lies the abhorrent idea of a desolate and reduced existence, disconnected from more-than-human relational lifecycles. As Stirling (2022) warns about how neoliberal education affects children’s temporal self-awareness, “growing up becomes a process not only of moving toward adulthood and therefore subjectivity but also. . .to sever relations with nonhuman worlds and view oneself as no longer entangled in common fates” (p. 39).

My daughter’s words challenge the tendency to understand temporality solely as progress or deterioration, and material decay and mortality as something undesirable. Such stories call for education to recognise and attend to the temporal complexities of contemporary childhoods. Researchers working through posthuman and new-materialist perspectives have suggested that children’s environmental experiences and imaginaries, especially connected to climate change and ecological disasters, might come to disrupt linear clock time in education (Carrizo et al., 2025; Da Rosa Ribeiro et al., 2023; Rooney and Blaise, 2023; Tammi et al., 2024). They propose that relational, materially grounded and nonlinear approaches to time resonate with the discordant, tangled and multidirectional environmental relationships of contemporary childhoods. Carrizo et al. (2025), for example, write about the temporalities of dust in early childhood education and propose that dust holds rich material sediments of various dissolving and decomposing bodies and materials across centuries, connecting “children and nonhumans to all forms of cosmological life and times” (p. 264). Through such material-temporal framings, the core of time becomes more-than-human relational movement, and conceptions of time become ontologically and ethically significant questions for education.

Understanding time through relational movement, underscores material vulnerability and porosity. As Neimanis and Walker (2014) propose, time becomes “a transcorporal stretching between present, future, and past” (p. 558). This perspective shifts us from linear conceptions of time to relational, hybrid and cyclical understandings, which resonate with Indigenous ontologies (Stirling, 2022). Such a shift, allows education to become attuned to the deep, situated ongoingness of children’s learning, relating, and becoming with landscapes and other beings, rather than merely prioritizing lofty educational goals (Rooney and Blaise, 2023; Stirling, 2022). Notions of relational movement and vulnerability can cultivate alternative kinds of temporal awareness, ethics, and future imaginings that recognise time as fleshy, immediate, thick and intensity-rich more-than-human worldings (Neimanis and Walker, 2014; Tammi et al., 2024). Consequently, engaging with the past, present, and future alongside children transforms into a slow and sticky traversing through multidirectional, rhizomatically dense material-temporal landscapes.

## Living temporalities: Ecological chronotopes in children’s augmented storying

### Kristiina Kumpulainen

How is time experienced in children’s encounters with ecological worlds? This piece develops the concept of *ecological chronotopes* to examine how ecological processes organise narrative time in children’s storying practices. Drawing on Bakhtin’s (1981) notion of the chronotope—the intrinsic connectedness of time and space in narrative—I explore how children’s narrative practices make perceptible the temporal rhythms already unfolding within ecological settings. Ecological chronotopes foreground how lived temporalities emerge through encounters with ecological processes such as seasonality, decay, regeneration, and multispecies interdependence.

The argument develops in dialogue with my ongoing research in Finland and Canada with children aged 6–8 years and their teachers, focusing on ecological imagination through augmented storying with local environments (see, e.g. Kumpulainen et al., 2023). I use the term *augmented storying* to refer to place-responsive narrative practices in which children layer digital elements onto material environments. Through these practices, children compose stories that do not simply represent ecological settings and children’s cultural worlds but unfold within their temporal rhythms. Ecological chronotopes thus refer to narrative–temporal configurations in which ecological processes organise lived time and render alternative temporalities experientially available.

Recent multispecies research has drawn attention to ontological instability in children’s encounters with more-than-human others, including what has been described as “flickering ontologies” (Renshaw et al., 2024), where the boundaries between the real and the possible momentarily blur. Building on this insight, I shift the analytic focus toward the temporal–spatial conditions through which such instability becomes structured and experienced. Ecological chronotopes direct attention to how narrative encounters with ecological processes intensify temporal awareness and open imaginative engagements with multiple temporal registers.

From this perspective, ecological chronotopes are not only narrative structures but also affective and material fields in which ecological temporalities become experientially intensified. Through them, processes such as duration, interruption, continuity, and transformation become perceptible in children’s encounters with ecological worlds. Ecological settings, whether forests, shorelines, urban wetlands, or school grounds, articulate temporal patterns that exceed linear progression and invite cyclical, recursive, and layered experiences of becoming. In this sense, ecological worlds do not merely host stories; they participate in teaching time.

This orientation resonates with Adam’s (1998) notion of “timescapes*”*, where temporality is rhythmic and embedded within ecological processes, and with Barad’s (2007) account of temporality as emergent through intra-action. Perspectives on more-than-human temporalities further illuminate this shift. Ingold (2011) conceptualises environments as ongoing processes of growth and correspondence, Haraway (2016) foregrounds “thickened” presents shaped by multispecies entanglement, and Tsing (2021) attends to precarious temporalities unfolding within damaged landscapes. Extending these perspectives, ecological chronotopes foreground how environments generate temporal orientations that children encounter and inhabit.

Childhood studies have long critiqued developmental models that position children primarily within future-oriented trajectories (Lee, 2001; Prout, 2004). Qvortrup (1994) further highlights how institutional temporalities structure generational relations. Ecological chronotopes offer a complementary perspective by situating children within broader ecological times that precede and exceed developmental timelines. In such contexts, children are not simply moving toward the future but are dwelling within layered temporalities shaped by ecological processes.

My research shows how augmented storying reveals the ways ecological settings reorganise narrative time. Within these practices, past ecological traces, present encounters, and imagined futures coexist in the same storied moments. As children move through environments and layer digital narratives onto material landscapes, ecological rhythms become perceptible. Digital mediation thus amplifies awareness of the layered temporalities already unfolding within ecological worlds.

Conceptually, ecological chronotopes contribute to theorizing temporality in childhood by decentring human developmental time and foregrounding ecological temporalities as formative. They provide an analytic lens for examining how environments participate in structuring lived time and how narrative mediates this encounter. Temporality, in this view, is neither solely imposed by institutions nor produced by individuals; rather, it emerges within ecological relations and is articulated through narrative practice.

In times marked by environmental precarity, attending to ecological chronotopes opens new possibilities for understanding how lived time is shaped by and responsive to more-than-human temporalities. By extending chronotopic theory beyond literary analysis into environmental and childhood literacy research, it can be shown how ecological processes themselves organise narrative time and how children’s storying practices render these temporal relations perceptible.

## Storying with mythical beings as slow pedagogy in environmental education

### Chin Chin Wong and Jenny Byman

Myths and folklore, such as spirits in nature and forest elves, are often used to engage children in exploring their community’s worldviews, traditions, and beliefs. Learning grounded in myths is suggested as a powerful means of opening transformative ways of relating, being in the world and re-imagining possible futures (Molloy Murphy, 2021). In our ethnographic study, we noticed that multiple temporalities and rhythms emerged when children (aged 7–9) engaged in storying activities with mythical beings, entangling with moments of hesitation, silences and slow embodied movements (Byman et al., 2025). Below, we engage with these temporal patterns of children and mythical beings with the theoretical perspectives of *slow pedagogy*.

Studies on slow pedagogy in environmental education emphasise the importance of integrating a “slow” temporal experience to deepen the understanding of place and ecological relationships (Clark, 2022). In our project “Riddle of the Spirit” in Finland, we engaged children (aged 7–8 years) in a primary school in lingering with global issues such as climate change with pre-modern Baltic Finnic myths about nature spirits. Baltic-Finnic myths are entangled with specific places, oral stories, landscapes, and the dynamic forces of the natural world across Karelia, Ingria, Finnish, and Sami traditions and lifeworlds. The children were invited into the world of mythical beings through an imaginative storyline: the thunderstorm spirit Ukko has lost the ability to control the weather. With other nature spirits, including the forest spirit Tapio and the water spirit Vetehinen, the children pondered the cause of this troublesome situation and engaged in playful activities that linked to the nature spirits’ worlds.

Our ethnographic approach links slow pedagogy and slow childhoods to more-than-human worlds of folklore. Slow pedagogy is not only about changing the ontological and epistemological focus in education; rather, it requires a “practical and systemic venture” (Dýrfjörð and Hreinsdóttir, 2025: 2) to challenge how we think about rhythm and linear clock-time in childhood. Slow time, or “uninterrupted time”, with more-than-human beings allows uncertainty and unruliness and encourages children to get deeply involved with places and landscapes, attune to environmental details and arouse wonder about ecological systems and issues climate change causes on these. In line with this, our research emphasised the importance of playful and open-ended pedagogical practices in creating chances for children’s deliberation and meaning-making about the stories, places and their materialities they encounter. In the storying activities, many children were deeply engaged with issues related to their local forest, expressing concerns about the garbage in the woods and how it could harm the nature spirits. Some children acted out how the spirits might see the local forest with anger, frustration and helplessness. Others created imaginative solutions, such as the forest spirit Tapio bringing firewood to the forest and bringing it back to life. These encounters all emerged when children moved with the mythical beings, the local environment and the material configuration of the Riddle of the Spirit project.

The temporality of children’s storying with mythical beings was characterised by shifting and dynamic relations of time. As Farquhar (2016) discusses temporal entanglement, we inhabit the intersection of various forms of time and must reconsider how multiple temporalities coexist in pedagogical practices of early childhood education. In our project, the myths of nature spirits were reimagined and transformed, fuelled by children’s curiosity, care and concerns about socio-ecological issues. Storying with mythical beings created movements comparable to “speculative spaces” (Byman et al., 2025), transcending linear time and allowing different layers of timespace to interact. Spontaneous embodied movements, “off-task-ness” and moments of hesitation were important speculative spaces for children to engage with issues related to natural forces and climate change, such as forest degradation and environmental pollution (Byman et al., 2025). These speculative spaces emerged from an active doing and a “becoming different” in relation to the worlds of nature spirits through open-ended activities that attended to lingering with the process itself, rather than focusing on a clear-cut outcome.

We therefore invite researchers and practitioners to recognise children’s slow rhythms and imaginative encounters with mythical beings as meaningful opportunities to unsettle dominant narratives and imagine alternative realities. In children’s stories, human exploitation, stewardship of natural resources, and ethical kinship are questioned and reimagined. Meanwhile, care comes to matter as a force for repairing earthly relations in our common world. Storying with myths bridges the world as it was, the world as it is, to the world as it could be and should be (Molloy Murphy, 2021). These practices invite speculative fabulations of just ecological relationships and caring worlds with the more-than-human.

## Time and embodiment in children’s community placemaking processes

### Jacqueline “Jacquie” Kociubuk

Self-care children, or children who spend time without direct caregiving adults’ supervision during out-of-school hours, are a common fixture in U.S.-based public libraries and one of my favourite phenomena to study in terms of their community placemaking processes. This group of children is varied in terms of their demographics, yet united in how they construct the public library from simply a community space into a place with meaning and value. As I have spent time exploring different facets of their placemaking processes, the ideas of embodiment (the connection between our physical bodies and minds) and temporality are increasingly an area of curiosity in my work.

The intertwining of embodiment, time, and place draws from a rich history of human geographic scholarship and complements childhood studies’ conversations around children’s bodily and temporal experiences in institutional and familial contexts. In seeing connections between these discipline-specific discourses, I find a need to put together foundational human geographic scholarship like Seamon’s (1980) time-space routines and place-ballets and Rose’s (1993) time-space embodiment with Prout (2000) and other childhood studies colleagues’ work on children’s physical bodies and the social construction of childhood. The conversation between these scholars, and additional insights from childhood geographers like Blundell (2016), has helped me better understand the importance of children’s temporality-related embodiment in community placemaking processes.

Looking first towards the geographers, for many self-care children, going to the library is part of the pattern of their days; exactly what Seamon (1980) describes as a time-space routine. The ebb and flow of children entering and exiting the library space coincides with library staff work habits creating what Seamon refers to as a “place-ballet”. However, in research I have conducted, I have observed that which children are allowed to have the library be part of their time-space routine and what activities they are allowed to engage in is tied up with their current age and/or adults’ perception of children’s maturity relative to their embodied appearance (other ties include racial and gender identity). For example, library policies set out a temporal bound around the age children are allowed into the space unattended, and library staff’s visual interpretation of how old a child (and their race or gender identity) influences whether certain behaviours are allowed (e.g. running is more permissible for a kindergartner than a teenager, more acceptable for a white girl than a Black African American boy).

Helping to make the connection from time-space routines to the physical body, Rose (1993) discusses embodiment, saying that we must acknowledge that when bodies do not fit the standard temporal experience or desires of “white bourgeois men” they are marginalised within geographic spaces (p. 77). My conversations with children (aged 8–18 years) echo this sentiment, with many of the participants describing how the library is a space for those much older or much younger than them as evidenced by the materials, toys, and decor that do not match their developmental stage or desired activities (e.g. plastic toy food, storytime rugs, quiet zones, newspapers). This contrasts with the library staff I talk to who enthusiastically show me various spaces they have designed within their libraries for differently-aged children; a disconnect hinting at how the design of children’s institutional spaces is frequently rooted in adult-centric notions of how childhood time should be spent at certain stages of development (Blundell, 2016; Prout, 2000).

Bringing these thoughts together with Prout (2000) and contemporaries’ ideas of an embodied and socially-constructed childhood, I conclude that children’s bodily experiences of the passage of time, and adults’ projection of developmental time, inform children’s community placemaking processes in multiple ways. As children progress through adolescence, their perceptions and valuation of spaces and places change with time (e.g. children’s area becomes babyish or boring). Further, their experiences with a space also change as adults respond to children’s advancement in age (e.g. older children are more of a threat or should know how to “behave”). Thus, I find I can no longer simply detach children’s experiences with a space from their physical bodies, because social constructions of childhood are so frequently shaped by physiologically-related time markers.

## Rural children’s experiences of time

### Layla Coleman

Within the United States rural spaces are characterised through open countryside, undeveloped spaces, and agricultural economies; landscapes peppered with small towns and villages under 2,500 people (Ratcliffe et al, 2016; Tieken and San Antonio, 2016). Long regarded as spaces that embrace a slower pace of life, common conceptualizations of rural time or country time can be traced back to the Roman age, where rurality was considered only in contrast to the town (Oyen, 2019). While these temporal conceptualizations are flawed and limited, Oyen proposes that the conventional view of rural life is still “an image of the past” (p. 192) with continuing assumptions that “the countryside occupies a different temporal register than the city” (p. 194). While Oyen (2019) argues that rural agency is silenced by perceived notions of rural time as slow time, as a child, my experience in the rugged and unpopulated space echoes these generalizations, particularly during summer and my early childhood experiences, where time felt ever unhurried.

The scenery of my childhood was filled with aging family farms, steep bluffs and coulees, and tangled new-growth forests, a backdrop ripe for a slow and unhurried rural childhood. Known as the Driftless Area, this topographically unique chunk of the upper Mississippi River valley (bypassed by the last glacial drift, some 12,000 years ago) culturally and temporally embraces the slogan: *Slower is Better*. While I can neither validate nor refute the value of slowness, my recent reflections on interviews conducted with children (ages 9–15) exploring their digital engagements made me wonder: do the children living in this rural region today experience their childhoods as slow? Do digital media influence rural children’s current experiences of time?

The lived experiences of rural children are distinct and shaped by the specific cultural, geographical, and social knowledges that children hold. Through work discussing the aspirations of and possibilities for rural youth, Tieken and San Antonio (2016) observe that rural contexts shape how youth see themselves, how they perceive the world, and how they form dreams and aspirations about who they might be. Research has also shown that children hold insight and awareness concerning rurality, able to identify both popular and familial discourses concerning rural life and relay the activities and experiences that construct their definitions of “being rural” (McCormack, 2002).

The specific experiences and discourses of rural children’s lives become even more interesting when we turn toward research considering children’s perceptions of time. In a study on preschool children’s perspectives on time, Dýrfjörð et al. (2023) note that young children measure time through specific activities, events, or experiences where “the sense of the duration of time passing is dependent on context rather than hours on a clockface” (p. 14). If young children are more likely to measure time in relation to their engagements, we can in turn expect the practices and materials situated within rural contexts to shape rural children’s experiences of time. As Tilhou (2021) notes in discussion of Maria, a young participant in Punch’s (2002) ethnographic work exploring rural children’s perceived spatialities, “the passage of time is shaped by [Maria’s] routine which is shaped by [Maria’s] culture, as the culture is shaped by land and agriculture there” (p. 48).

Rural children’s lives are no longer lived only in the unpopulated spaces of rural landscapes, but also within the unique digital cultures and engagements that they are privy to (Burnett et al., 2014). During my own interviews with urban and suburban youth about their social media use and creative digital engagements, many discussed their lack of personal time and the “rushing around” that their lives require. While my childhood was filled with slow and unhurried time, I wonder how current digital realities have shifted experiences of time for rural children today. Do they still experience the kind of time that stretches and lingers as I did? Perhaps the relentless pace of digital participation makes rural experiences of expansive time even more desirable.

## Youth and techno-temporal spaces

### Melanie Wong

By attending to time and space, we can identify and explore the unique moments in which meaning is created (Pahl, 2014). Rather than viewing time as a uniform flow that overlooks individual actions, it can be understood as shaped by shared memories and personal subjectivities, enabling us to question and challenge the dominant epistemologies that constrain these perspectives. In today’s increasingly post-digital world, the affordances of digital technologies allow youth to navigate a variety of learning spaces that are both school sanctioned and interstitial, or non-sanctioned (Wong, 2021). This fluid movement in and in-between learning spaces reveals how youth often engage in practices that diverge from school-sanctioned or institutional activities. Temporality in these interstitial, non-sanctioned learning spaces differs markedly from school-sanctioned environments, where institutional and colonial structures govern and restrict how students’ time is organised. The rigid structures of schools, dictated by bells and prescribed instructional hours mandated by government policy, allocate youth’s time with precision, down to the second.

Compton-Lilly (2016a) has asserted that “time acts as a constitutive dimension of people’s experiences that significantly affects how people make sense of their worlds” (p. 576). In the digital realm, how youth choose to fill their time highlights what they value but also how they are making sense of their world. Notably, when interviewing my research participants, who are school aged (ages 5 to 17) students in Canada, their answers are illuminating when it comes to temporality in the lifeworlds of youth in a digital age. These participants shared about their interstitial learning experiences, including time devoted to accessing and watching unknown experts on YouTube Howcasts to inform a school-sanctioned assignment (Wong, 2021) and Rap Battling in the in-between spaces to counter school-sanctioned activities (Wong, 2019). Although this research did not address time but rather space, these findings illustrate the ways that youth are choosing to allocate their time, and more importantly, the findings illuminate how temporality in interstitial learning spaces differ significantly from school-sanctioned learning spaces. In these interstitial learning spaces, students have agency to allocate time as they choose.

In my ongoing exploration of emerging technologies and technology-enhanced classrooms, I continue to notice the ways that youth push back against their institutional experiences by reconfiguring learning spaces into more playful ones. Other scholars have also looked at this notion of play in digital spaces (Edwards et al., 2026; Işıkoğlu et al., 2023; Stephen et al., 2020). Yet, digital play and time continues to be under-researched. When engaging in digital play, students’ conception and allocation of time, likely differs significantly from school-sanctioned spaces where research has documented how youth spend time engaging in digital play such as virtual worlds or video games (see Grimes, 2021; Pettersen et al., 2022). Hence, in these interstitial learning spaces, they are allocating their time in activities that differ significantly from school-sanctioned time allocation. Further to this, how we perceive time as adults differs from a child’s understanding and perception of time. We are socialised at a young age into certain practices of time (e.g. school starts at this time, play is during recess, during school hours we are engaging in school-sanctioned activities). But in interstitial and therefore unstructured learning spaces, time is not restricted and youth have more agency with how they structure their time allocation. Hence, I argue that it is important to consider how students perceive and use their time in interstitial learning spaces, as these contexts often provide flexible, self-directed opportunities that contrast with the structured, time-regulated routines of school-sanctioned learning environments.

## Temporalities and children’s media encounters

### Rebekah Willett

This reflection draws on several studies over the past 5 years which included interviews with children (ages 4–15) and their parents in the US about family media practices. Time is a frequent topic in these conversations. Talking through daily routines, we often hear about allocations of screentime for children and daily routines that involve co-viewing. Family media practices are also part of weekly and seasonal routines: Friday movie nights are common in families, parents have lie-ins on weekends by giving their children more screentime on Saturdays, and screentime rules are more relaxed in summers when time is less structured.

“Clock time” also emerges in discussions with children about their digital making activities (e.g. creating art, videos, code, music). We hear about children using media differently depending on the time available. Part of digital making is playing around with the possibilities of technologies—having Canva create AI generated images that respond to particular prompts, for example. These “messing around” activities (Ito et al., 2009) require small amounts of time and can be done in interstitial moments; and, as one child shared, they require little creative effort. However, when immersed in a complex digital project, investment of time is necessary. Importantly, children indicate that these deeper media encounters allow them to be more creative compared with moments they might spend on a platform or technology as a way to “pass the time”. In their theorization of “living literacies”, Pahl and Rowsell (2020) stress the importance of children having time to experience a “what if state of mind”, particularly in relation to creative activities: “What if I take this sheet of cardboard and use it to make a hydraulic arm? . . . making entails these kinds of fluid, continuous, passion-driven practices that mix reason with sensation” (p. 21). Clock time is connected with different types of digital practice and varying “sensations” of creative making.

However, “clock time” is only one temporal experience framing children’s media encounters. The *feeling* of time is also important to consider. In their analyses of young children’s sense of time in preschool settings, Dýrfjörð et al. (2023) argue that there is “an embodied sense of time that is subjective, relational and situational” (p. 1). In our discussions with children, we hear how time can “fly” when immersed in a digital making project and can “creep” when a problem arises and frustration ensues. In one of our studies, a child talked about being on their tablet playing Minecraft “all day” one summer day; however, this did not mean she played Minecraft non-stop from morning until night; rather, it felt like the whole day because she was playing Minecraft in between all her other summer activities.

Finally, children express a sense of time passing and feelings of nostalgia in relation to their media practices. Children’s reflections align with Compton-Lilly’s (2016b) temporal discourse analysis of children’s literacy lives. Compton-Lilly writes, “temporal comparisons not only contribute to how families made sense of ongoing experiences, but also to the possible futures they envisioned” (p. 42). Children share recollections about mistakes they made as a “newbie” in a game or online space, positioning themselves as now older and wiser. As children revisit their earlier digital creations, they have memories of what it felt like when they were younger and struggling with what now seems simple, sometimes even nostalgic for times in their past. Looking back also reflects moments of placemaking—children might remember a particular time and place from when they were younger and engaged in a particular digital creation. Memories of making are often infused with relational components—a connection to something they were interested in at that moment of making, a problem a sibling helped them solve, a project they worked on with friends, an old piece of technology that is now handed-down to younger family members, a Scratch club at their former school or a nearby library. If we as researchers are interested in understanding children’s encounters with media and technologies, we need to consider the affordances and constraints of clock time and the more experiential sense of time through which children are making sense of their worlds.

## The privilege of pausing: Children reflecting on media and reading experiences

### Markéta Supa

Children’s contemporary media and reading lives are increasingly governed by the imperative to consume and progress quickly. Whether in print, digital or multimodal forms, engagement with text, image and sound is shaped by a logic of acceleration. Algorithms and digital platforms encourage more frequent, faster and larger-volume interaction with content, and educational assessments often measure reading in terms of proficiency levels and numbers of books completed. These linear metrics neglect the diverse ways children engage with media and literature and leave little room for reflection on their authentic experiences. Taking time to pause and reflect offers a form of resistance to these external pressures. Instead of always moving forward, children can turn inward and responsively explore their own thoughts and feelings.

We might first associate such pauses with education or parenting, but they can also be intentionally cultivated within childhood research. In our phenomenologically guided conversations with children (ages 9–12) in Czechia, we used tangible props and open-ended prompts to support reflection on their media and reading experiences (see Supa et al., 2025; Kuzmičová and Supa, 2024; Kuzmičová et al., 2022). While talking with and listening to our participants, I repeatedly witnessed their capacity and delight in returning to past experiences of reading, viewing, imagining, playing, creating, and wondering. As van Manen (1997) noted, lived experiences gain meaning when gathered through memory and reflection in the acts of dwelling in time.

Importantly, children reflected not only on what they had once done, but also on how these experiences had felt and how they continue to matter in the present and for the future. In this way, our research conversations opened up shared spaces and moments in time for “remembering with analysis” (Epstein, 2003: 2) opening a distinct temporal horizon for both children and researchers. Disturbing “the illusion of linear time” (Pomerantz and Knight, 2025: 307), pausing to reflect invited children to stay with experiences long enough for multiple meanings to emerge. They could slow down, re-feel, re-think, and re-imagine their past, present, and future media and reading experiences, while momentarily resisting a culture of moving quickly from one act of consumption to the next, from reading to reading “better” or “more”. While doing so, they made sense not only of these experiences, but also of themselves, others, and the world around them.

Being with children in moments of reflection, I felt honoured and grateful. Although we (researchers) may have our own aims and goals—whether related to research or to children’s learning—we should, phenomenologically speaking, at least momentarily bracket them. This makes it possible for childhoods and adulthoods to dialogically co-exist in shared understanding (Sheppard, 2025). However, the temporality of awareness is essential. Reflection should not aim at constant self-consciousness, but at allowing new understandings to settle, fade, and re-emerge within the flow of living (Gadamer, [1960] 2004; Holquist, 2002). As Gadamer reminds us, every act of understanding repositions the past within the present and opens possibilities for the future. Reflection thus mediates between two “interacting temporal modes” (Bjerrum Nielsen, 2016: 1); it unfolds within chronological life while continually reworking meaning in non-linear time. Here, children’s media and reading experience remains fluid and in dialogic relation with what was, what is, and what follows (Woodfall and Zezulkova, 2016), as well as with the world of others.

Yet any reflective pausing, and listening, does not occur in isolation from the outside world, because a person has not only “a history, but also a context” (Bjerrum Nielsen, 2016: 5). Reflection belongs to children themselves, but the capacity to pause cannot be understood as purely individual. It is learned, socially mediated, and culturally shaped, rather than innate or universal (Lindholm, 2018). Pausing to reflect is therefore a privilege. It is unevenly accessible across children and communities and impacted by wider conditions of time equity (Bettencourt et al., 2025). In media- and text-saturated worlds, creating contexts in which every child can *claim the time and space* to pause and reflect is thus a matter of equality, inclusion, and social justice—a commitment to which research with children can actively contribute.

## On listening: Stories, play, imagination, and time

### Harini Rajagopal

Children come to understand the rhythms of time—past, present, and future—gradually, through deeply human aspects like imagination and storying, in relation with themselves and the world. I wonder about how we (adults, teachers, systems) slow down time to make space for these learnings. *How does our cultural habit of imposing a sense of urgency to our daily lives —often reinforcing power imbalances and disconnecting us from the need to wonder, linger, and reflect —ironically distract us from the true urgency of addressing racial, social, ecological, and cultural injustices, of being well with each other and the world?*

My two school-aged children often look through their baby photos on my phone with wonder and nostalgia. As my phone throws up memories, their fingers swipe across the screen; “When was that?” they ask, giggling as their younger selves look back at them—chubby cheeks and curled toes frozen in time. We share stories and each image feels like a tender echo of moments wrapped in warmth, and stories told over and over.

The stories cradle them in a quiet kind of delight

a reminder that they were once tiny.

Each image, a whisper from yesterday,

cradled in the palm of now—

proof that they were magic once,

and still are.

With my children and the children I research with, often racialised and marginalised young people, stories—family narratives, picture books, or imaginative play—function as both a bridge and a pillar. Playful storying (Dyson, 2021; Rajagopal, 2025) invites children to travel outside the here and now, becoming windows, mirrors, and sliding doors (Bishop, 1990) that construct alternative realities. Stories serve as pillars because children learn about what happened when, and *why* it matters, and the ability to imagine futures grounded in past understandings. Through stories and play, children reassemble past experiences, reframe future possibilities, and trace their present (Archibald, 2008; Rajagopal and Anderson, 2025; Wohlwend, 2023). Imagination, then, becomes a rehearsal space that transcends time.

This synergy between imagination and stories shapes not only how children learn but how they live in the now. Yet, in a world that is always moving forward in time, children are told to urgently prepare for becoming something other than what they are. Moving them upwards across stages and grades, propels milestones, lessons, practices, assessments, and ways of doing within society and schools. When notions of precarious times (Malone et al., 2017) and turbulent times (Arar et al., 2024) flood our inboxes, and discourses of achievement gaps (Sealey-Ruiz, 2022) and lack of readiness (Tager, 2017) inundate our educational systems, Bayo Akomolafe reminds us, “times are urgent, let us slow down” (Akomolafe and Benavides, 2016). Imagination, stories, and play invite such slowing down.

As educators and researchers participating in children’s lives, slowing down becomes not a function of speed but of awareness, intention, and attunement. Slowing down becomes not about getting answers but about questioning our questions, facing the invisible and looking at the unresolved. Listening to children creatively (Rajagopal and Kendrick, 2023; Rinaldi, 2006) as they story, play, and imagine helps us to move from witness to with-ness, and to be with children and the world in relation. This pausing, noticing, wondering, and listening becomes not only communication but also a critical imaginative space for community building, decolonizing knowledge, and countering contingency. Speculative listening also opens up practices of presencing (Nxumalo and Bozalek, 2021), oriented towards building liberatory worlds for human and more-than-human coexistents and interrupts the marginalisation of certain people, onto-epistemologies and relations from specific places. Relatedly, taking up “arts of awareness” (Tsing, 2021) and inquiring about children’s imagination become provocations for generating innovative, relational, and emergent methods to attend to the complexity of childhoods.

Wrapping up this piece and returning to the poetic magic of all children, I let their questions, stories, art, experiences, and emotions shape the flow of inquiries and the learning that unfolds from it. Stories of bike rides, bad haircuts, புத்தாண்டு celebrations, paper constructions, leaves and sticks, and drawings of family weave emotional meaning and ways of being and becoming. As neoliberal and settler colonial logics press urgency, slower attentive practices and methodologies can enhance childhood studies (Nelson, 2019) and envision more ethical ways of engaging with lives and learning.

## Concluding thoughts

Our collective reflections indicate that temporal lenses are particularly valuable for highlighting onto-epistemological approaches to research with children that centres on the diversity and complexity of their experiences. Through slow reading and careful revisiting of data across multiple projects, we have become more aware of children’s engagement with multi-layered timescapes. Children’s literacy, ecological, placemaking and media experiences do not have to be rushed or driven by linear and essentialist notions of development, progress, and time. Rather, we recognise that they can be slow, uneven, and multidirectional. For children, time is reflected in their embodied changes and encounters with growth, ageing and finitude that shape how they experience and understand the world and how others respond to them. In turn, when temporal conditions support rather than constrain children’s attentiveness to themselves and others, children can reflect, imagine alternatives, create, and resist. In doing so, they can access and explore a variety of ways of living, being and learning in relation to their own as well as the world’s past, present, and futures.

By carefully exploring children’s experiences and interactions beyond isolated moments, and by taking into account their identities, histories, and contexts, we may develop a deeper sensitivity to childhood and time. We also recognise that time is a privilege and that it is not distributed evenly. Engaging in dialogic reflection with our international colleagues over an extended period, while revisiting our past research through new lenses, has been a privilege for all of us. Being with children and listening to them, whether in research or everyday situations, is another such privilege. When approached with empathy and responsiveness, it can help us move from witnessing to with-ness. Our contribution therefore lies not only in the outcome, but in the process itself. As such, the collective both demonstrates and advocates for slow, collaborative and caring childhood scholarship and, by extension, for time equity.
